# Engineered *Saccharomyces cerevisiae* for the *De Novo* Biosynthesis of (−)-Menthol

**DOI:** 10.3390/jof8090982

**Published:** 2022-09-19

**Authors:** Xueqin Lv, Xuan Zhou, Jun Ma, Mengrui Tao, Yanfeng Liu, Jianghua Li, Guocheng Du, Long Liu

**Affiliations:** 1 Key Laboratory of Carbohydrate Chemistry and Biotechnology, Ministry of Education, Jiangnan University, Wuxi 214122, China; 2 Science Center for Future Foods, Jiangnan University, Wuxi 214122, China

**Keywords:** menthol, *De Novo* synthesis, D-limonene, *Saccharomyces cerevisiae*

## Abstract

Menthol, a high-value commodity monoterpenoid chemical, holds an important market share commercially because of its distinct functions. The menthol on the market mainly originates from plant extraction, which is facing challenges such as the seasonal fluctuations and long growth cycle of plants. Therefore, this study attempted to realize the *de novo* synthesis of menthol through microbial fermentation. First, through heterologous expression and subcellular localization observation, a synthetic route from glucose to (−)-menthol was successfully designed and constructed in *Saccharomyces cerevisiae*. Then, the mevalonate (MVA) pathway was enhanced, and the expression of farnesyl diphosphate synthase (*ERG20*) was dynamically regulated to improve the synthesis of D-limonene, a key precursor of (−)-menthol. Shake flask fermentation results showed that the D-limonene titer of the recombinant strain reached 459.59 mg/L. Next, the synthesis pathway from D-limonene to (−)-menthol was strengthened, and the fermentation medium was optimized. The (−)-menthol titer of 6.28 mg/L was obtained, implying that the *de novo* synthesis of menthol was successfully realized for the first time. This study provides a good foundation for the synthesis of menthol through microbial fermentation.

## 1. Introduction

Menthol, a cyclic monoterpene alcohol with well-known cooling characteristics, has become one of the world’s most widely used flavoring additives and has been applied in a variety of consumer products ranging from candy to oral care products, cigarettes, and even over-the-counter medicinal products [1,2]. The global market demand for menthol can reach 30,000 t per year [1], of which ~70% is extracted from mint plants (mostly spearmint, peppermint, or corn mint), and the other 30% is mainly produced through chemical synthesis or semisynthesis [3,4]. The seasonal fluctuation and long growth cycles of plants are the challenges encountered in the production of menthol. Recently, with the development of synthetic biology and its advantages in the synthesis of natural compounds derived from plants [5,6,7], the green biological manufacture of menthol by breaking traditional production methods through synthetic biology has attracted the extensive attention of researchers [8,9].

In a recent development, menthol was produced in the laboratory by using a cell-free one-pot biotransformation strategy [9]. In short, the researchers developed a one-pot biosynthesis method for menthol isomers with pulegone as the substrate using the extracts of recombinant *Escherichia coli* incorporating the “ene”-reductase encoding gene NtDBR (double-bond reductase) from *Nicotiana tabacum* and two menthone dehydrogenases encoding the (−)-menthone: (−)menthol reductase (MMR) and (−)-menthone: (−)neomenthol reductase genes from *Mentha piperita* [9]. Additionally, some related studies included the synthesis of myrcene, a monoterpene, as a starting material for menthol production by metabolically engineered *E. coli* [10]. However, the *de novo* biosynthesis of menthol has not been realized, and the research on the synthesis of menthol through microbial fermentation remains in its infancy.

Menthol has three chiral centers, implying eight possible different stereoisomers, among which (−)-menthol is the most preferred isomer [11,12]. Therefore, in this study, we focused on the biosynthesis of (−)-menthol. The *de novo* synthesis of (−)-menthol with *Saccharomyces cerevisiae* as the starting strain was successfully realized using a variety of metabolic engineering strategies. First, through the heterologous expression and subcellular localization observation of eight enzymes involved in menthol synthesis, a synthetic route from glucose to (−)-menthol was designed and constructed in *S. cerevisiae*. Then, the mevalonate (MVA) pathway was enhanced, and the expression of farnesyl diphosphate synthase (*ERG20*) was dynamically regulated with the glucose-sensing promoter HXT1 to improve the synthesis of D-limonene, a key precursor of (−)-menthol. The titer of D-limonene reached 459.59 mg/L in shake flask fermentation. On this basis, the synthesis pathway from D-limonene to (−)-menthol was strengthened, and in combination with fermentation medium optimization, the *de novo* synthesis of (−)-menthol was successfully realized for the first time with the titer of 6.28 mg/L. All of the results of this work lay a foundation for the efficient bioproduction of (−)-menthol.

## 2. Materials and Methods

### 2.1. Medium and Cultural Conditions

The *E. coli* JM109 strain required for the genetic experiments was cultured at 37 °C in Luria–Bertani medium containing 100 μg/mL ampicillin. *S. cerevisiae* CEN.PK2-1C was selected as the starting strain, and plasmid DNA or polymerase chain reaction (PCR) fragments were transformed using the LiAc/PEG/ssDNA method to construct the desired recombinant strains, which were screened on yeast nitrogen base (YNB) lacking the appropriate nutrients at 30 °C. The seed suspension used for shake flask fermentation was cultured in yeast extract peptone dextrose (YPD) medium. After 16–20 h of culture at 30 °C, the prepared seed suspension was inoculated into a 500 mL shake flask containing 50 mL of medium with an inoculum size of 2%. The media used for menthol production were soya peptone medium (fermentation medium I) and inorganic salt medium (fermentation medium II). Soya peptone biphasic medium was used for limonene synthesis. Supplementary Table S1 lists the composition and content of the media.

### 2.2. Construction of Plasmids and Recombinant Strains

Table 1 and Table S2 list all the plasmids and strains used in this study. The (−)-limonene synthase gene (*LimS*) and truncated LimS (*tLimS*) from *Mentha spicata*; (−)-limonene-3-hydroxylase (L3H) gene; the NADPH cytochrome P450 reductase (CPR1) gene from *Stevia rebaudiana*; the (−)-trans-isopiperitenol dehydrogenase (IPDH) gene, (−)-isopiperitenone reductase (IPR) gene, MMR gene, and (+)-pulegone reductase gene (PGR) from *Mentha x piperita*; and the steroid isomerase gene (KSI) from *Pseudomonas putida* were synthesized after codon optimization by Genewiz (Suzhou, China). PrimeSTAR Max Premix DNA polymerase (TaKaRa, Shiga, Japan) was used for gene amplification following the recommended protocol. All terminators or promoters were amplified using the genome of *S. cerevisiae* 288C as the template, and three plasmids (PY13, PY14, and PY15 and PY26) were used to construct gene expression cassettes.

In this study, the truncated HMG-CoA reductase (*tHMG1*), *ERG10*, *ERG13*, *ERG12*, *ERG8*, *ERG19*, and *IDI* genes were cloned from the genomic DNA of *S. cerevisiae* 288C for the construction of recombinant strains to enhance the MVA pathway flux. The overexpression of *ERG20^ww^* (*ERG20^F96W^-^N127W^*), a mutant of *ERG20*, was used to reduce farnesyl diphosphate (FPP) drain toward sterol biosynthesis. The genes of the (−)-menthol synthesis and MVA pathways were inserted into the corresponding plasmid through fusion PCR. All fragments and plasmids were verified by sequencing before yeast transformation. Homologous recombination was performed in *S. cerevisiae* by using the CRISPR–Cas9 or CRISPR–loxP system to achieve genes knockout or gene expression cassette integration [14]. YNB medium with specific amino acid deficiency was used to cultivate the obtained engineering strains.

### 2.3. Two-Phase Flask Cultivation for D-Limonene Production

Precultured cells (OD600 = 0.2) were inoculated into 50 mL fermentation medium I with 5 mL of dodecane for D-limonene extraction. Shake flask fermentation was performed at 30 °C and 220 rpm for 96 h. After fermentation, all bacterial liquid was collected and centrifuged at 4 °C and 6000× *g* for 10 min. D-limonene was obtained in the upper organic phase.

### 2.4. Confocal Imaging

Following the specific experimental requirements, enhanced green fluorescent protein (EGFP) was fused with the LimS, L3H, CPR, IPDH, IPR, KSI, PGR, and MMR fragments. Then, the fusion fragments were inserted into the corresponding plasmid to obtain the recombinant strains. All the strains were cultured in 2 mL of YPD medium at 30 °C and 220 rpm for 16–20 h in a 15 mL shaker tube. The samples were excited by using the 488 nm laser line of the argon ion laser line of a He–Ne laser. Fluorescence emissions were detected using the EGFP spectral detector sets BP 505–525. Finally, Leica TCS SP8 software and ImageJ software (NIH) were used for image analysis.

### 2.5. Fermentation and Extraction for Menthol Production

For the cultivation of (−)-menthol synthesis, fermentation medium II was cultivated for 96 h under the conditions of 30 °C and 220 rpm. A total of 1.5 mL of fermentation broth was taken and centrifuged at 12,000× *g* for 2 min to separate the supernatant and the precipitate. A total of 500 μL of ethyl acetate was added to the supernatant, and the supernatant was shaken for 2 min. The precipitate was resuspended with 1 mL of ethyl acetate with 0.5 mm glass beads, and the cells were broken by using MP Fastprep-24 5G (Santa Ana, CA, USA). Then, the samples were centrifuged for 2 min at 12,000× *g* after 20 min at room temperature. A total of 200 μL of the organic phase of the supernatant sample and 400 μL of the organic phase in the precipitate sample were taken for mixing. The resulting solution can then be used for the analysis of (−)-menthol production.

### 2.6. Gas Chromatography–Mass Spectrometry Analysis

Samples (ethyl acetate treated mixture) were added to anhydrous sodium sulfate for dewatering. A total of 1 μL of the organic phase was analyzed on a TSQ 8000 instrument (ThermoFisher, Waltham, MA, USA). The production of (−)-menthol was analyzed using a Rxi-5ms column (30 m × 0.25 mm × 0.25 μm, Restrk, Bellefonte, PA, USA). In this system, the detector was set to be in the positive ion polarity mode. Gas chromatography–mass spectrometry (GC–MS) was performed with a scan time of 0.2 s and a mass range of 35–350 amu. The GC oven temperature was initially held at 70 °C for 8 min and ramped to 150 °C at the rate of 5 °C/min for 3 min. Then, the temperature was increased to 280 °C at the rate of 2 °C/min for 3 min. The carrier gas was helium. The injector was maintained at 280 °C. The ion source temperature was 300 °C.

### 2.7. Statistical Analysis

All of the experiments in this study were conducted at least three times independently. The results were expressed as mean ± standard deviation. Statistical significance is indicated as * for *p* < 0.05 and ** for *p* < 0.01.

## 3. Results and Discussion

### 3.1. Construction of the (−)-Menthol De Novo Biosynthetic Pathway

The biosynthesis of monoterpenes by microbial hosts has attracted increasing attention [15,16]. However, relevant studies on (−)-menthol have been unremarkable mainly because isoprenone isomerase has remained unidentified. The 2018 discovery that KSI from *P. putida* could exercise the function of isophthalone isomerase provided a new prospect for (−)-menthol biosynthesis [5]. Considering that *S. cerevisiae* is an ideal platform organism for monoterpenoid biosynthesis, which can effectively provide the biosynthetic precursor geranyl diphosphate (GPP) [17,18], we selected *S. cerevisiae* as the host strain in this study.

**Figure 1:**
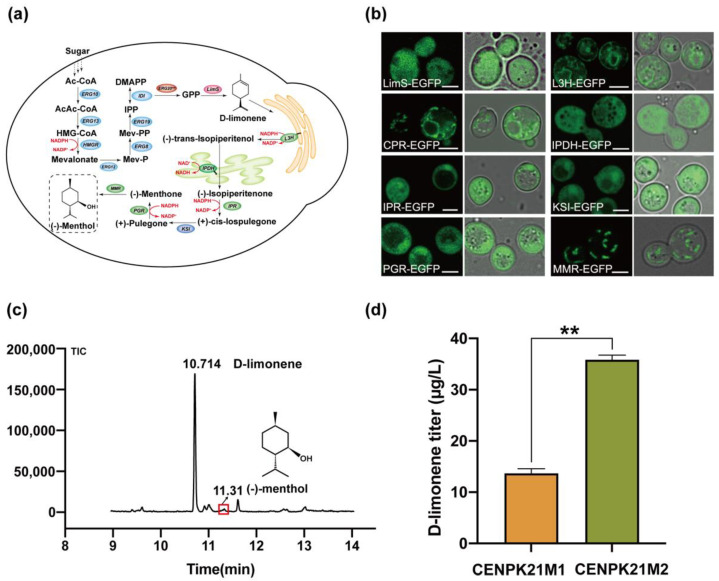
Schematic of pathway remodeling and compartmentalization of D-limonene and (−)-menthol: (**a**) illustration of the (−)-menthol biosynthesis pathways in *S. cerevisiae*; (**b**) subcellular localization of the eight genes involved in (−)-menthol biosynthesis; (**c**) GC–MS diagrams of D-limonene and (−)-menthol; and (**d**) titer of the intermediate product D-limonene in the CENPK21M1 and CENPK21M2 strains (Statistical significance is indicated as ** for *p* < 0.01).

First, we designed a synthetic route to menthol synthesis starting from glucose to give access to (−)-menthol by introducing eight exogenous enzymes into cells. These enzymes included LimS from *M. spicata*; CPR1 from *S. rebaudiana*; L3H, IPDH, IPR, PGR, and MMR from *Mentha x piperita*; and KSI (Figure 1a). Subsequently, we generated a series of recombinant strains expressing a C-terminal EGFP fused to LimS, L3H, CPR, IPDH, IPR, KSI, PGR, and MMR to observe the expression and distribution of exogenous enzymes in cells. Through laser scanning confocal microscopy, we observed obvious green fluorescence signals in each recombinant cell (Figure 1b). These observations indicated that these exogenous enzymes could be normally expressed in *S. cerevisiae*. Notably, L3H–EGFP, CPR–EGFP, and MMR–EGFP exhibited specific organelle localization characteristics (Figure 1b). This result suggests that the synthesis pathway of (−)-menthol may involve the participation of multiple organelles.

For the acquisition of a genetically stable *S. cerevisiae* strain that could synthesize (−)-menthol, we integrated *LimS*, *L3H*, *CPR*, *IPDH*, *IPR*, *KSI*, *PGR*, and *MMR* into the genome of *S. cerevisiae*, thus generating CENPK21M1. Shake flask fermentation with fermentation medium I showed that (−)-menthol was not detected in strain CENPK21M1. The full-length *LimS* from spearmint has a long plastidial targeting sequence, and the catalytic efficiency of *tLimS* is improved by removing this targeting sequence [19,20]. Surprisingly, in our study, replacing *LimS* with *tLimS* resulted in the successful detection of trace amounts of (−)-menthol in the obtained strain CENPK21M2 (Figure 1c). Given that D-limonene is a key precursor of (−)-menthol, we speculate that the cells have a very weak limonene synthesis ability that resulted in extremely low menthol contents. To verify our hypothesis, we analyzed the D-limonene titer in the above shake flask fermentation experiment, and the results showed that the D-limonene titer was only 13.69 mg/L in CENPK21M1 and 35.83 μg/L in CENPK21M2 (Figure 1d). These findings indicate that the low concentration of D-limonene is indeed an important factor limiting menthol synthesis. Therefore, the next experiment focused on the biosynthesis of D-limonene.

### 3.2. Enhancement of the MVA Pathway to Improve D-Limonene Synthesis

**Figure 2:**
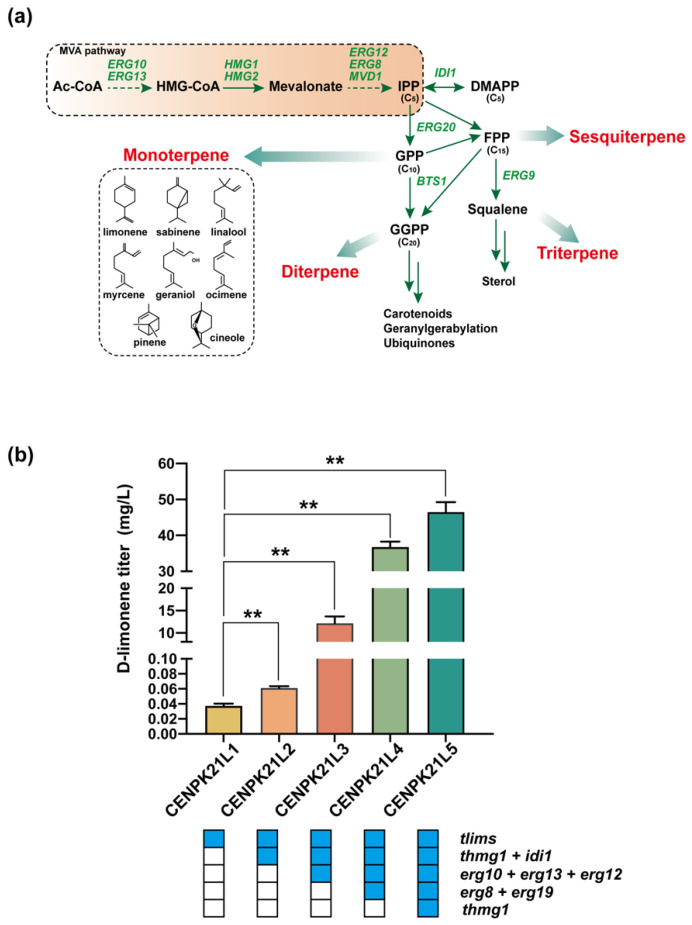
Stepwise increase in D-limonene production through the enhancement of the MVA pathway of monoterpene synthesis: (**a**) metabolic pathways for the production of various terpenes; and (**b**) D-limonene accumulation in engineered strains. Blue boxes represent gene overexpression. The limonene titer was only 53.14 µg/L when only the *tHMG1* and *IDI* genes were strengthened; reached 12.18 mg/L after further strengthening *ERG10*, *ERG13*, and *ERG12*; and reached 36.77 mg/L after all seven genes of the MVA pathway were enhanced. After another *tHMG1* copy was added, the limonene titers reached 46.96 mg/L (Statistical significance is indicated as ** for *p* < 0.01).

IPP and DMAPP produced from the MVA pathway are the essential building blocks for the synthesis of terpenoids, including D-limonene [21] (Figure 2a). Therefore, we attempted to strengthen the MVA pathway to obtain a recombinant strain with strong D-limonene synthesis ability. However, monoterpenes are highly toxic to many microorganisms [1,9]. The minimum inhibitory concentration of D-limonene for *S. cerevisiae* is 0.44 mM [22]. In this study, we adopted two strategies to address the toxic effect of D-limonene on cells. One is extraction fermentation, an effective strategy that can reduce the toxicity of monoterpenes [22], and the other is uncoupling cell growth and product synthesis. To achieve this goal, we selected the galactose-inducible *GAL* promoter (P*_GAL_*) to drive the expression of related genes in the MVA pathway.

We first used P*_GAL_* to induce the expression of *tLimS* to construct the D-limonene synthesis pathway in *S. cerevisiae* CEN.PK2-1C to monitor the change in the synthetic capacity of cells for D-limonene. We found that the D-limonene titer of the obtained strain CENPK21L1 was 36.76 µg/L. Then, strain CENPK21L2 was generated through the overexpression of truncated *tHMG1* and dimethylallyl diphosphate isomerase (*IDI1*) under the control of P*_GAL_* to supply sufficient amounts of the precursors IPP and DMAPP, and the D-limonene titer increased to 53.14 µg/L (Figure 2b). On this basis, the expression of *ERG10*, *ERG13*, and *ERG12* was strengthened by P*_GAL_*, resulting in strain CENPK21L3, which had a D-limonene titer that had increased by nearly 230 times to 12.18 mg/L (Figure 2b). After the remaining two MVA pathway genes *ERG8* and *ERG19* were enhanced to generate CENPK21L4, the D-limonene titer further increased to 36.77 mg/L (Figure 2b). Considering that HMG-CoA reductase is the key rate-limiting enzyme of the MVA pathway, we added another copy of *tHMG1* into CENPK21L4 to obtain strain CENPK21L5. The D-limonene titer of strain CENPK21L5 increased to 46.96 mg/L (Figure 2b), which was almost 1278 times that of strain CENPK21L1, without obvious changes in cell growth. All the above results indicated that enhancing the MVA pathway can significantly improve the ability of the cells to synthesize D-limonene.

### 3.3. Dynamic Regulation of ERG20 Gene Expression

**Figure 3:**
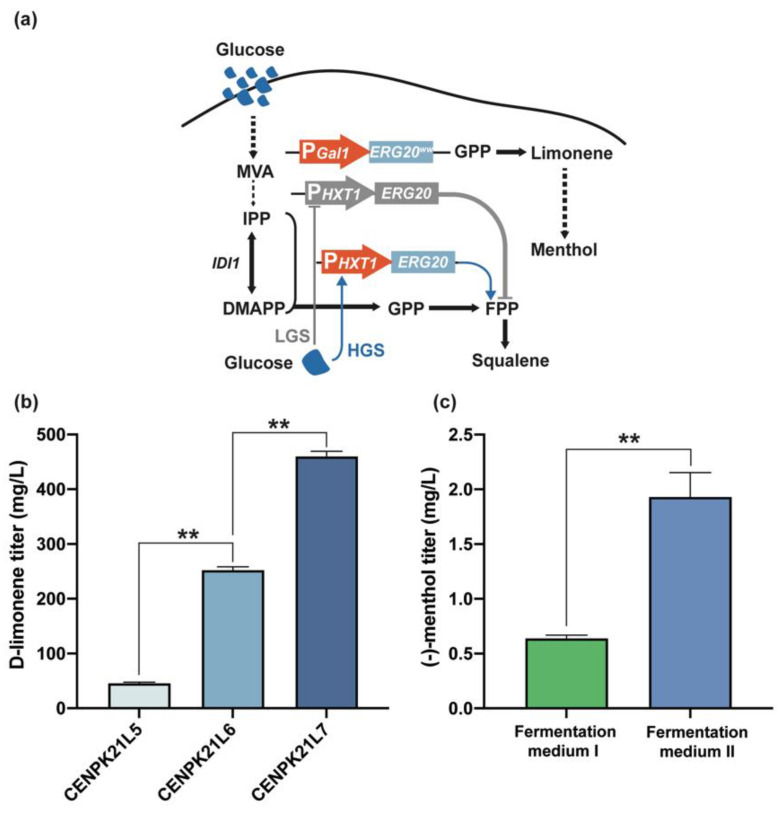
Dynamic regulation of *ERG20* gene expression. (**a**) Dynamic regulation strategies for (−)-menthol synthesis. The native promoter of chromosomal *ERG20* was replaced with the *HXT1* promoter. Arrows represent positive regulation, and blunt-ended lines represent negative regulation. LGS: low glucose, HGS: high glucose. (**b**) Dynamic regulation of *ERG20* by P*_HXT1_* increased the limonene titer by 3.5 times. Increasing the copy number of *ERG20^ww^* and *tLims* could further increase the limonene titer to 459.59 mg/L. (**c**) Optimized fermentation medium is more conducive to (−)-menthol production than nonoptimized fermentation medium. Statistical significance is indicated as ** for *p* < 0.01.

In *S. cerevisiae*, the heterologous expression of LimS can catalyze GPP formation through the condensation of DMAPP with IPP to synthesize D-limonene (Figure 1a). However, under the catalysis of endogenous *ERG20*, GPP can form FPP, which can dimerize into squalene for further entry into the ergosterol synthesis pathway [21]. Thus, *ERG20* is indispensable because ergosterol is the essential component of cell membranes for maintaining permeability and fluidity [23]. Through the dynamic expression of *ERG20* and the constitutive expression of *ERG20^ww^* (ERG20F96W-N127W), the monoterpene geraniol can be produced by geraniol synthase from the substrate GPP [24,25]. Therefore, we used the inducible promoter P*_GAL_* to overexpress *ERG20^ww^* in strain CENPK21L5 while using the glucose-sensing promoter P*_HXT1_* to regulate the expression of *ERG20* dynamically to increase the flow of GPP into D-limonene synthesis in the later stage of fermentation [24] (Figure 3a). Shake flask fermentation revealed that the D-limonene titer of CENPK21L6 was further increased by 3.5 times to 213.08 mg/L (Figure 3b). With the addition of another copy of *ERG20^ww^* and *tLimS*, the D-limonene titer of the obtained strain CENPK21L7 reached 459.59 mg/L (Figure 3b).

**Figure 4:**
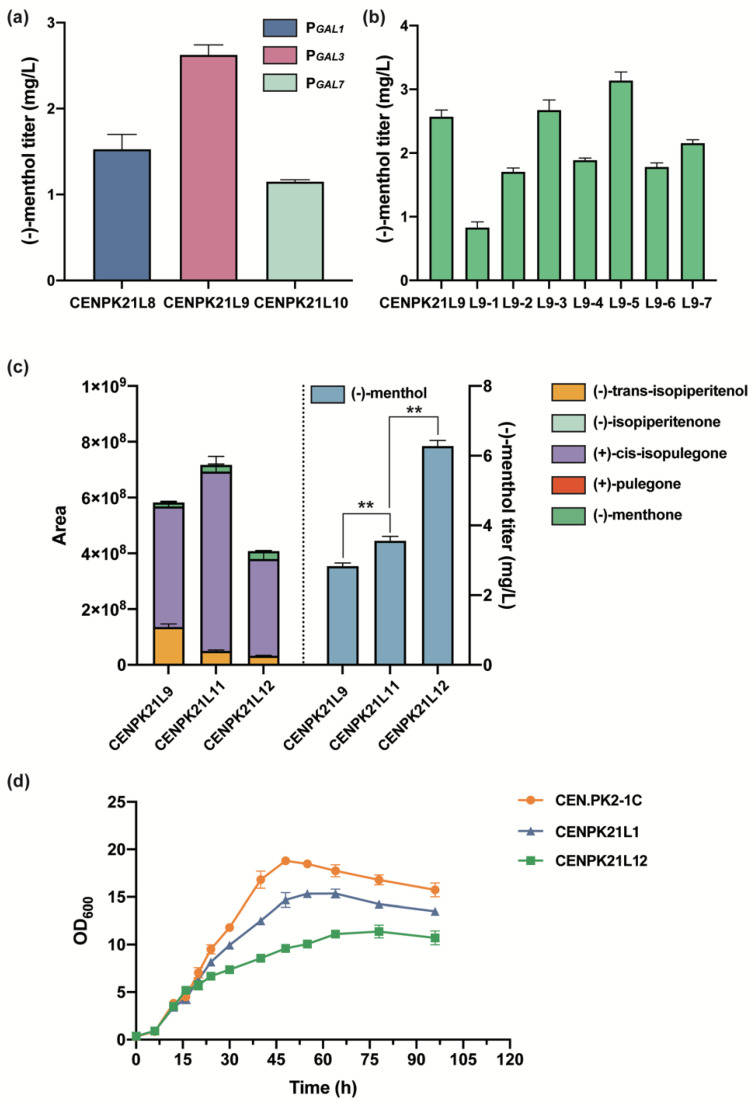
Optimization of the downstream pathway for (−)-menthol synthesis: (**a**) effect of different promoters of MMR on (−)-menthol titer; (**b**) effect of the enhanced expression of menthol downstream pathway genes by plasmids on (−)-menthol titer; (**c**) accumulation of different intermediates and corresponding (−)-menthol titers in menthol synthesis (Statistical significance is indicated as ** for *p* < 0.01); and (**d**) comparison of the OD_600_ of different strains.

To investigate the ability of the engineered strain to synthesize (−)-menthol, we introduced L3H, CPR, IPDH, IPR, KSI, PGR, and MMR sequentially into strain CENPK21L7. We thus generated CENPK21L8, which had a (−)-menthol titer that reached 0.64 mg/L (Figure 4a). Inspired by the fact that fermentation media rich in inorganic salts are beneficial for the production of the sesquiterpene artemisinic acid by *S. cerevisiae* [26], we attempted to optimize the fermentation medium. Our results showed that fermentation medium II, which was composed of 75 g/L sucrose, 20 g/L peptone, 15 g/L (NH4)2SO4, 11 g/L succinic acid, 10 g/L glucose, 0.15 g/L methionine, 8 g/L KH2PO4, 6 g/L MgSO4·7H2O, 2 g/L sodium glutamate, 0.72 g/L ZnSO4·7H2O, 20 mg/L uracil, 12 mL/L vitamin, 10 mL/L microelement, and 1.5 mL/L biotin, can significantly increase the production of (−)-menthol to 1.93 mg/L (Figure 3c). However, this titer is still very low.

### 3.4. Optimization of the Downstream Pathway for (−)-Menthol Synthesis

Previous studies have shown that MMR competes with KSI for the substrate (+)-cis-isopulegone, thus leading to the production of other by-products that affect menthol production [8]. For the optimization of the expression intensity of MMR and KSI, KSI was initiated by the strong promoter *GAL1* based on strain CENPK21L8, and the original *GAL1* promoter of MMR was replaced with the two promoters *GAL3* and *GAL7* with different intensities to obtain strains CENPK21L9 and CENPK21L10, respectively. The results showed that when MMR was expressed with the *GAL3* promoter with the lowest expression intensity, the (−)-menthol titer reached 2.62 mg/L (Figure 4a). For the determination of the rate-limiting step of the heterologous pathway, the *L3H*, *L3H* and *CPR*, *IPDH*, *IPR*, *KSI*, *PGR*, and *MMR* genes in the menthol downstream pathway were enhanced by the free expression of plasmids based on the CENPK21L9 strain to obtain strains L9-1–L9-7. The results showed that (−)-menthol increased only after IPDH and KSI were strengthened and that the (−)-menthol titers of L9-3 and L9-5 reached 2.67 and 3.14 mg/L, respectively (Figure 4b).

We further examined the intermediate metabolites (−)-trans-isopiperitenol, (−)-isopiperitenone, (+)-cis-isopulegone, (+)-pulegone, and (−)-menthone between limonene and menthol by using GC–MS. The contents of the above intermediate metabolites were generally low. However, the accumulation of (−)-trans-isopiperitenol and (+)-cis-isopulegone was higher than that of (−)-isopiperitenone, (+)-pulegone, and (−)-menthone (Figure 4c). Combined with the above analytical results of using plasmids to enhance (−)-menthol heterologous pathway gene expression, strain CENPK21L11 was obtained by adding an extra copy of IPDH to the genome of strain CENPK21L9, (−)-trans-Isopiperitenol content significantly reduced, and the (−)-menthol titer reached 3.56 mg/L (Figure 4c). On this basis, strain CENPK21L12 was obtained by adding one copy number of KSI; (+)-cis-isopulegone was significantly reduced; and the (−)-menthol titer reached 6.28 mg/L (Figure 4c), which was 122% higher than that of strain CENPK21L9.

Notably, the maximum OD_600_ values of the (−)-menthol-producing strains were less than 12, and high (−)-menthol titers were associated with low maximum OD_600_ values (Figure 4d) likely because the P*_GAL_* promoter will lead to the leakage expression of the target genes. On the other hand, in addition to the final product (−)-menthol, the synthetic pathway of (−)-menthol contains nearly 10 other intermediate metabolites of monoterpenoids, which are easily extracted in the organic phase such that extraction fermentation cannot be performed. These monoterpenes, which are extremely toxic to host cells [1,9], eventually inhibit cell growth. Therefore, in future research, reducing the toxicity of (−)-menthol and its intermediate metabolites to host cells is the primary problem to be addressed in constructing a cell factory for efficient (−)-menthol synthesis.

## 4. Conclusions

By expressing eight exogenous enzymes, we constructed a route for the *de novo* synthesis of (−)-menthol in *S. cerevisiae*. The *de novo* synthesis of menthol was realized for the first time through this route in combination with other strategies, such as the enhancement of the MVA pathway, the dynamic regulation of *ERG20*, and the optimization of the fermentation medium. Therefore, our work lays a foundation and provides a new direction for the construction of cell factories for the efficient synthesis of (−)-menthol.

## Figures and Tables

**Table 1:** *Saccharomyces cerevisiae* strains used in this study.

Strain	Host Strain	Description	Source
CEN.PK2-1C			Lab work
CENPK21M1	CEN.PK2-1C	△gal80: P*_GAL10_*-LimS, △YPRCδ15c::CPR1-P*_GAL1,10_*-L3H, △208a:IPDH-P*_GAL1,10_*-IPR, △1622b::KSI-P*_GAL1,10_*-PGR, △1021b: P*_GAL1_*-MMR	This study
CENPK21M2	CEN.PK2-1C	△gal80: P*_GAL10_*-*tLims*, △YPRCδ15c::CPR1-P*_GAL1,10_*-L3H, △208a:IPDH-P*_GAL1,10_*-IPR, △1622b::KSI-P*_GAL1,10_*-PGR, △1021b: P*_GAL1_*-MMR	This study
CENPK21L1	CEN.PK2-1C	△gal80: P*_GAL10_*-*tLims*	This study
CENPK21L2	CENPK21L1	△1014a:*tHMG1*-P*_GAL1,10_*-*IDI1*	This study
CENPK21L3	CENPK21L2	△416d:*ERG10*-P*_GAL1,10_*-*ERG13*-P*_GAL7_*-*ERG12*	This study
CENPK21L4	CENPK21L3	△1309a:*ERG19*-P*_GAL1,10_*-*ERG8*	This study
CENPK21L5	CENPK21L3	△1309a:*ERG19*-P*_GAL1,10_*-*ERG8*-P*_GAL7_*-*tHMG1*	This study
CENPK21L6	CENPK21L5	△911b:P*_GAL1_*-*ERG20^ww^*, P*_ERG20_*-*ERG20*::P*_HXT1_*	This study
CENPK21L7	CENPK21L6	△SAP115b:*ERG20^ww^*-P*_GAL1,10_*-*tLims*	This study
CENPK21L8	CENPK21L7	△YPRCδ15c:CPR1-P*_GAL1,10_*-L3H, △208a:IPDH-P*_GAL1,10_*-IPR, △1622b:KSI-P*_GAL1,10_*-PGR, △1021b: P*_GAL1_*-MMR	This study
CENPK21L9	CENPK21L8	P*_GAL1_*-MMR:P*_GAL3_*	This study
CENPK21L10	CENPK21L8	P*_GAL1_*-MMR:P*_GAL7_*	This study
L9-1	CENPK21L9	PY26-L3H	This study
L9-2	CENPK21L9	PY26-L3H-CPR1	This study
L9-3	CENPK21L9	PY26-IPDH	This study
L9-4	CENPK21L9	PY26-IPR	This study
L9-5	CENPK21L9	PY26-KSI	This study
L9-6	CENPK21L9	PY26-PGR	This study
L9-7	CENPK21L9	PY26-MMR	This study
CENPK21L11	CENPK21L9	△308a: P*_GAL1_*-IPDH	This study
CENPK21L12	CENPK21L11	△308a: P*_GAL1_*-KSI	This study

Notes: YPRCδ15c, 208a, 1622b, 1021b, 1014a, 416d, 1309a, 911b, SAP115b and 308a were integration locus of chromosomal [13].

## Data Availability

Not applicable.

## Supplementary Materials

The following supporting information can be downloaded at: https://www.mdpi.com/article/10.3390/jof8090982/s1, Table S1: Different media composition and content. Table S2: Plasmids used in this study.
