# Supplementary material for: Engineered Saccharomyces cerevisiae for the De Novo Biosynthesis of (−)-Menthol
Source: J Fungi (Basel). 2022 Sep 19;8(9):982. doi: 10.3390/jof8090982 (PMC9503987; doi:10.3390/jof8090982)
Supplement: Supplementary file 1 [file jof-08-00982-s001.zip › jof-1911259-supplementary.pdf]

Supplementary Materials

Table S1. Different media composition and content

	Composition	Content
Fermentation medium I	Soybean peptone	50 g/L
	Glucose	25 g/L
	Sucrose	25 g/L
	K_2HPO4	0.6 g/L
	Glycerol	25 mL/L
Fermentation medium II	Sucrose	75 g/L
	Peptone	10 g/L
	Sodium glutamate	2 g/L
	$(NH_4)_2SO_4$	15 g/L
	KH_2PO_4	8 g/L
	$ZnSO_4 \cdot 7H_2O$	0.72 g/L
	$MgSO_4 \cdot 7H_2O$	6 g/L
	Succinic acid	11 g/L
	Uracil	20 mg/L
	Methionine	0.15 g/L
	Vitamin solution	12 mL/L
	Trace metal solution	10 mL/L
	Liquid paraffin	10 mL/L
	Glycerol	5 mL/L

Note:

Vitamin solution contained 62.5 mL/L biotin solution (1mg/ml), 1.25 g/L calcium pantothenate, 1.25 g/L nicotinic acid, 31.25 g/L myo-inositol, 1.25 g/L thiamine, 1.25 g/L pyridoxal, and 0.25 g/L p-aminobenzoic acid.

Trace metal solution contained 10.2 g/L $ZnSO_4 \cdot 7H_2O$, 0.5 g/L $MnCl_2 \cdot 4H_2O$, 0.75 g/L $CuSO_4 \cdot 5H_2O$, 0.47 g/L $CoCl_2 \cdot 6H_2O$, 0.56 g/L $Na_2MoO_4 \cdot 2H_2O$, 3.84 g/L $CaCl_2 \cdot 2H_2O$, 5.12 g/L $FeSO_4 \cdot 7H_2O$, 15 g/L EDTA.

Biotin solution, 1g biotin was dissolved into 1L of ethanol and 4N-sulfuric acid mixtures (1:1)

Table S2. Plasmids used in this study

Plasmids	Description	Source
PY13	Amp, HIS3, CEN/ARS, *E. coli*-*S. cerevisiae* shuttle vector	Lab work
PY14	Amp, TRP1, CEN/ARS, *E. coli*-*S. cerevisiae* shuttle vector	Lab work
PY15	Amp, LEU2, CEN/ARS, *E. coli*-*S. cerevisiae* shuttle vector	Lab work
PY15-erg20^{ww}-tLims	PY15 derivate, △P_{TEF1}:: Erg20^{ww}-$P_{GAL1,\ 10}$-tLims	This study
PY14-L3H-CPR1	PY14 derivate, △P_{TEF1}:: CPR1- $P_{GAL1,\ 10}$-L3H	This study
PY13-IPDH-IPR	PY13 derivate, △P_{TEF1}:: IPDH- $P_{GAL1,\ 10}$-IPR	This study
PY13-KSI-PGR	PY13 derivate, △P_{TEF1}:: KSI- $P_{GAL1,\ 10}$-PGR	This study
PY13-MMR	PY13 derivate, △P_{TEF1}:: P_{GAL1}-MMR	This study
PY26-L3H	PY26 derivate, △P_{TEF1}:: P_{GAL10}-L3H	This study
PY26-L3H-CPR1	PY26 derivate, △P_{TEF1}:: CPR1- $P_{GAL1,\ 10}$-L3H	This study
PY26-IPDH	PY26 derivate, △P_{TEF1}::P_{GAL1}-IPDH	This study
PY26-IPR	PY26 derivate, △P_{TEF1}:: P_{GAL10}-IPR	This study
PY26-KSI	PY26 derivate, △P_{TEF1}::P_{GAL1}-KSI	This study
PY26-PGR	PY26 derivate, △P_{TEF1}::P_{GAL10}-PGR	This study
PY26-MMR	PY26 derivate, △P_{TEF1}::P_{GAL3}-MMR	This study
